# Novel Characterization of Lymphatic Valve Formation during Corneal Inflammation

**DOI:** 10.1371/journal.pone.0021918

**Published:** 2011-07-07

**Authors:** Tan Truong, Eda Altiok, Don Yuen, Tatiana Ecoiffier, Lu Chen

**Affiliations:** Center for Eye Disease and Development, Program in Vision Science, School of Optometry, University of California, Berkeley, California, United States of America; Johns Hopkins University, United States of America

## Abstract

Lymphatic research has progressed rapidly in recent years. Though lymphatic dysfunction has been found in a wide array of disorders from transplant rejection to cancer metastasis, to date, there is still little effective treatment for lymphatic diseases. The cornea offers an optimal site for lymphatic research due to its accessible location, transparent nature, and lymphatic-free but inducible features. However, it still remains unknown whether lymphatic valves exist in newly formed lymphatic vessels in the cornea, and how this relates to an inflammatory response. In this study, we provide the first evidence showing that lymphatic valves were formed in mouse cornea during suture-induced inflammation with the up-regulation of integrin alpha 9. The number of corneal valves increased with the progression of inflammatory lymphangiogenesis. Moreover, we have detected lymphatic valves at various developmental stages, from incomplete to more developed ones. In addition to defining the average diameter of lymphatic vessels equipped with lymphatic valves, we also report that lymphatic valves were more often located near the branching points. Taken together, these novel findings not only provide new insights into corneal lymphatic formation and maturation, but also identify a new model for future investigation on lymphatic valve formation and possibly therapeutic intervention.

## Introduction

Accompanying the blood circulation, the lymphatic network penetrates most tissues in the body and plays critical roles in many functions, including immune surveillance, body fluid homeostasis, and fat and vitamin absorption. A wide array of diseases and conditions has been found to be associated with lymphatic dysfunction, such as cancer metastasis, transplant rejection, inflammation, and lymphedema [1], [2], [3], [4], [5]. However, to date, there is still little effective treatment available for lymphatic disorders.

The cornea of the eye offers an ideal site for lymphatic research [2], [6], [7]. As a transparent tissue, it is naturally devoid of any vasculatures at adult age. Nonetheless, lymphangiogenesis (LG, the development of new lymphatic vessels) can be induced in this tissue after an inflammatory, infectious, traumatic, or chemical insult. One great advantage of using the cornea for lymphatic investigation is that it is both easy and straightforward to evaluate newly formed lymphatic vessels in this tissue since there are no pre-existing or background vessels to consider. Experimental data generated from corneal LG studies are therefore assured of a high degree of accuracy and reliability.

Anatomically, the lymphatic network differs from that of blood circulation in that it is a unidirectional channel rather than a circuit. Interstitial fluid, macromolecules, and leukocytes collected from tissues first enter blind-ended lymphatic capillaries before they travel through collecting lymphatic vessels and return to the venous circulation via the thoracic or right lymphatic duct [1]. To prevent backflow in the unidirectional channels, lymphatic valves are formed in collecting lymphatic vessels. These valves are necessary to overcome opposing pressure gradients and move lymph along the lymphatic network in a stepwise manner [8]. Currently, it still remains a mystery whether newly formed lymphatic vessels in the cornea are equipped with the valves, and how their formation correlates with corneal inflammatory LG, which is the focus of this study.

LG is a complex process involving the interaction of lymphatic endothelial cells with the extracellular matrix through integrin cell surface receptors, which are a diverse family of heterodimeric cell surface transmembrane glycoproteins formed by the association of α and β subunits [9], [10], [11]. The specific roles of integrins in corneal LG are still largely unknown. Previous work from us and other researchers have demonstrated that integrin α1 and α5 are involved in corneal inflammatory LG and transplant immunity [12], [13], [14], [15]. Studies outside the eye have also shown that integrin α9 (Itga-9) knockout mice die shortly after birth because of severe systemic lymphatic deficiency [16]. A more recent study has reported that Itga-9 is expressed on mouse lymphatic valves during development, and its deficiency leads to valve malformation and retrograde lymph flow [17]. To this stage, there has been no study linking Itga-9 to lymphatic vessels in the ocular surface.

In this paper, we have shown, for the first time, Itga-9 expression is up-regulated during corneal inflammation. The number of Itga-9^+^ lymphatic valves increases as corneal LG progresses, and most valves are located near the lymphatic branching points. Furthermore, our results indicate that there is a maturation process for corneal lymphatic valves, which vary morphologically from incomplete to complete ones. These novel findings not only provide a new concept in understanding corneal lymphatic formation and maturation, but may also reveal a new target for therapeutic intervention of corneal LG and related diseases. We anticipate this study will offer a new model system to investigate inflammatory lymphatic valve formation and regulation. Future studies employing this new model may provide novel insights into a number of lymphatic disorders occurring outside the eye as well.

## Materials and Methods

### Mice and anesthesia

Normal adult 8–12 week old male BALB/c mice (Taconic Farms, Germantown, NY) were used in the experiments. All mice were treated according to ARVO Statement for the Use of Animals in Ophthalmic and Vision Research and a protocol approved by the Animal Care and Use Committee, University of California, Berkeley. Mice were anesthetized using a mixture of ketamine, xylazine, and acepromazine (50 mg, 10 mg, and 1 mg/kg body weight, respectively) for each surgical procedure, and all efforts were made to minimize suffering.

### Induction of corneal inflammatory lymphangiogenesis

The suture-induced inflammatory LG model was used to induce the growth of new lymphatic vessels into the cornea, as described previously [18]. Briefly, three 11–0 nylon sutures (AROSurgical, Newport Beach, CA) were placed into the stroma of central corneas 

without penetrating into the anterior chamber. Sutures were left in place and whole-

mount corneas were harvested at 3, 7, and 14 days post-surgery. The experiments were repeated twice with a total of 6 mice in each group of study.

### Reverse transcriptase and semi-quantitative PCR

The experiments were performed as described previously [12]. Briefly, total RNA was extracted and purified from normal and inflamed corneas 14 days after suture placement with an RNAeasy mini-kit from Qiagen (Valencia, CA). Reverse transcription was performed using the SuperScript® VILO™ cDNA synthesis kit from Invitrogen (Carlsbad, CA). PCR was performed with the PCR mastermix from Promega (Madison, WI). Primer sequences were: mouse Itga-9, forward 5′-TGATCAATATAAAAGGCTTACATTTAT-3′, reverse 5′-CTGATGCTGTTCTCT

TCCT-3′; mouse GAPDH, forward 5′-ACCACAGTCCATGCCATCAC-3′, reverse 5′-TCCACCACCCT GTTGCTGTA3′. For semi-quantitative analysis, NIH Image J software was used for normalization and analysis of the band intensity. The experiments were repeated 3 times.

### Immunohistochemical assays with epifluorescence or confocal microscopy

The experiments were performed according to our standard protocol [19], [20]. Briefly, freshly excised whole-mount tissues were fixed in acetone for immunofluorescent staining. Samples were sequentially incubated with purified rabbit-anti-mouse LYVE-1 (Abcam, Cambridge, MA) and goat-anti-mouse Itga-9 antibodies (R&D Systems, Minneapolis, MN), which were visualized by FITC-conjugated donkey-anti-rabbit and Cy3-conjugated donkey-anti-goat secondary antibodies, respectively. Double-stained samples were covered with Vector Shield mounting medium (Vector Laboratories, Burlingame, CA) and examined by an AxioImager M1 epifluorescence deconvolution microscope with AxioVision 4.8 software (Carl Zeiss AG, Göttingen, Germany), or a LSM 510 Meta/NLO Axioplan confocal microscope with LSM AxioImager software (Carl Zeiss AG). Focal areas running along the length of the LYVE-1^+^ vessels that were LYVE^−^ Itga-9^+^ were identified as areas of valve formation. The total number of lymphatic valves per cornea was quantified for each time point.

### Statistical analysis

Data are expressed as the mean ± SEM. Statistical analysis was performed by Student *t* test for PCR analysis or one-way ANOVA for time course study of valve counts. Prism software (GraphPad, La Jolla, CA) were used with *P*<0.05 considered significant.

## Results

### Itga-9 expression is up-regulated during corneal inflammation

**Figure 1:**
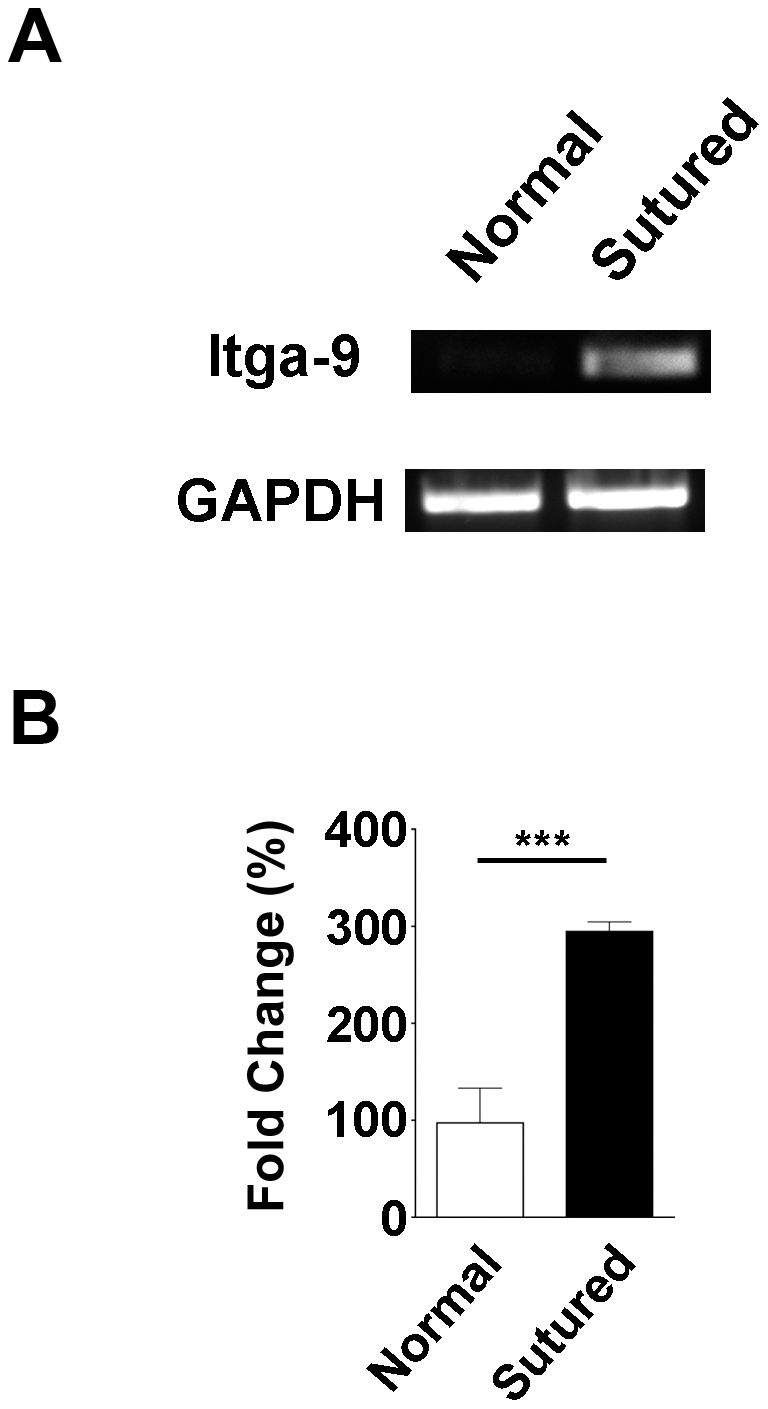
Itga-9 expression is increased in inflamed cornea. (**A**) Representative micrographs from semi-quantitative RT-PCR analysis showing that Itga-9 expression is significantly increased in 2 week post-sutured corneas compared with normal control. (**B**) Summarized data from 3 repetitive experiments. ***P<0.001.

It is known that Itga-9 labels lymphatic valves, and its up-regulation mediates lymphatic valve formation in developing non-ocular tissues [17]. We first set out to examine whether Itga-9 expression was also up-regulated during corneal inflammatory LG. To approach this, standard suture placement model was employed and the expression of Itga-9 was compared between normal and inflamed corneas 14 days after suture placement. At this time point, inflammatory LG has reached its peak and the cornea is already invaded by a large amount of newly formed lymphatics [21]. As shown in Figure 1, Itga-9 expression in the sutured corneas was significantly higher than in normal controls (***P<0.001).

### Itga-9 is expressed on newly formed lymphatic valves in inflamed corneas

**Figure 2:**
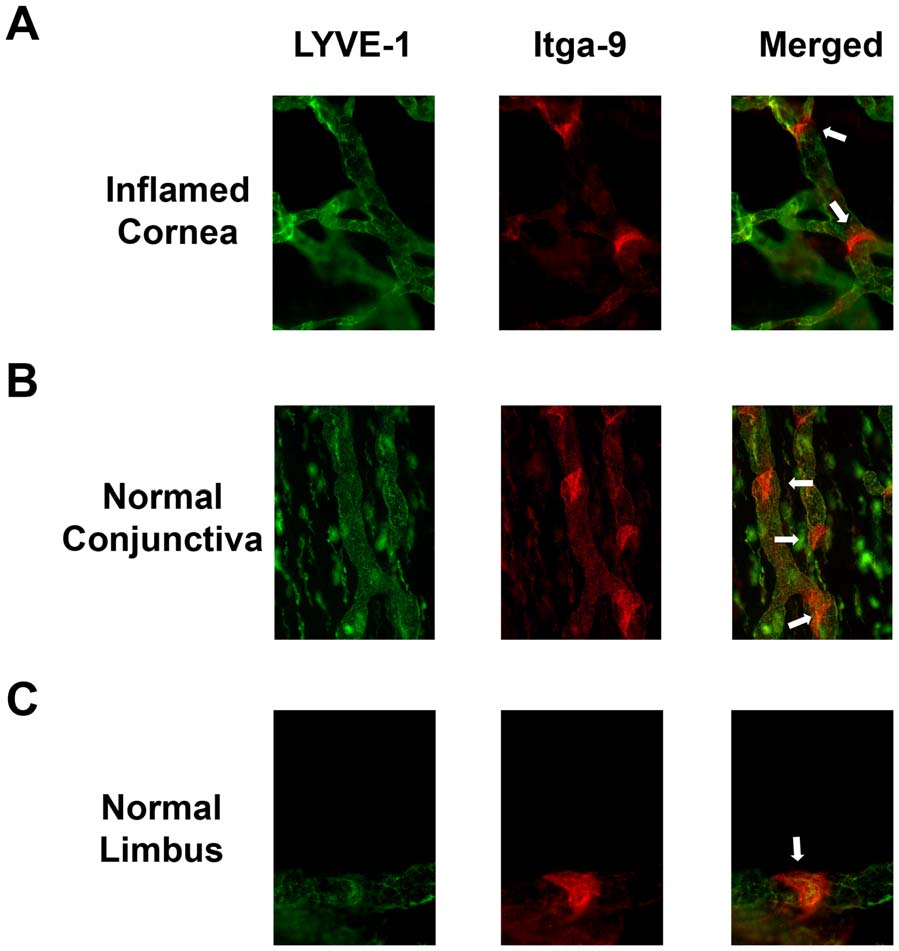
Itga-9 is expressed on lymphatic valves in inflamed cornea and normal conjunctiva and limbi. Representative micrographs demonstrating Itga-9 expression on luminal valve leaflets (arrows) of LYVE-1^+^ lymphatic vessels in inflamed corneas 2 weeks after suture placement (**A**), and normal conjunctiva (**B**) and limbus (**C**). Itga-9: red; LYVE-1: green. Original magnification: 200 X (**A** and **B**) and 400 X (**C**).

To further investigate the expressional pattern of Itga-9 in the inflamed corneas, we next 

performed immunofluorescent microscopic assays on sutured corneas 14 days post-surgery. As demonstrated in Figure 2A, Itga-9 was highly expressed on luminal valve leaflets of LYVE-1^+^ lymphatic vessels. The expression of Itga-9 was also detected on LYVE-1^+^ lymphatic walls at a lower level. The expression of Itga-9 on corneal lymphatic valves was further validated by an additional assay on normal conjunctiva (Figure 2B), which is known to be endowed with collecting lymphatic vessels [22] and can thereby serve as a positive control tissue for the valve staining method. Itga-9^+^ valves were observed in LYVE-1^+^ normal limbal lymphatic vessels as well, as shown in Figure 2C.

### Time course of lymphatic valve formation during corneal inflammatory lymphangiogenesis

**Figure 3:**
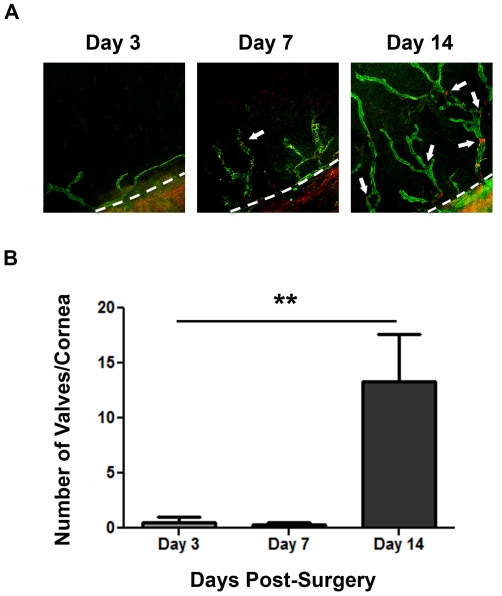
Time course of lymphatic valve formation during corneal inflammation. (**A**) Representative micrographs demonstrating the increase of lymphatic valves in sutured corneas with the progression of corneal inflammatory lymphangiogenesis. Itga-9: red; LYVE-1: green. Original magnification: 50 X. Dashed lines: demarcation between the cornea and conjunctiva. (**B**) Summarized data from repetitive experiments showing lymphatic valve quantification at day 3, 7, and 14 after suture placement. **P<0.01.

Having determined that lymphatic valves are present on newly formed lymphatic vessels in the cornea, we next examined the time course of lymphatic valve formation during corneal inflammatory LG. Whole-mount corneas at Day 3, 7, and 14 days after suture placements were subjected to series of immunofluorescent microscopic assays using specific antibodies against LYVE-1 and Itga-9. As presented in Figure 3A, the number of lymphatic valves increased as corneal LG proceeded. While minimal number of valves were found in lymphatic vessels in Day 3 and Day 7 corneas, a considerable amount of valves were detected in Day 14 samples. This trend reflecting the time course of lymphatic valve formation in inflamed corneas was summarized and presented in Figure 3B (**P<0.01).

### Lymphatic valves at different developmental stages in inflamed corneas

**Figure 4:**
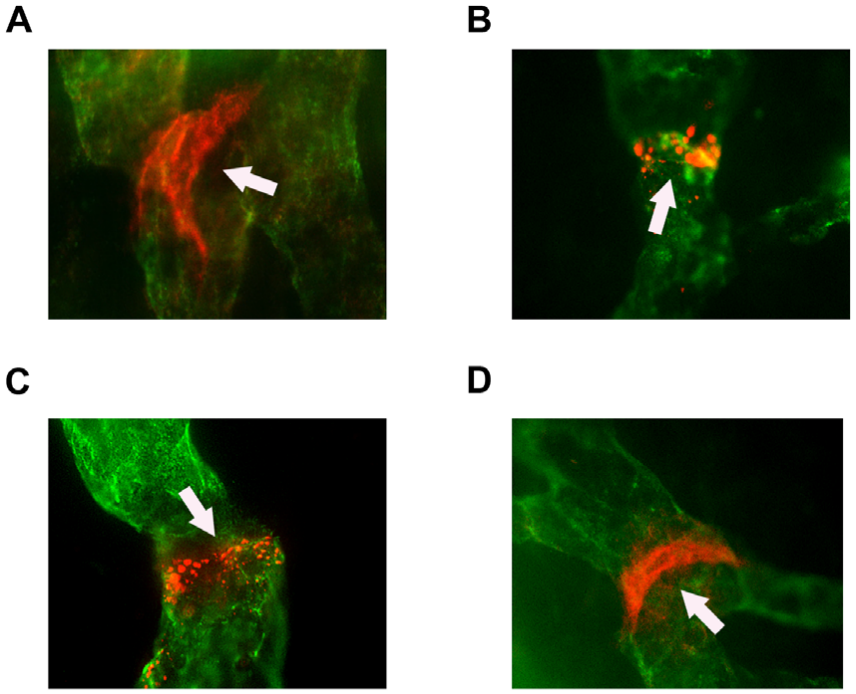
Morphologically distinct stages of lymphatic valve formation in inflamed cornea. Representative micrographs demonstrating a mature lymphatic valve in normal conjunctiva (**A**) and various stages of lymphatic valves in inflamed corneas 2 weeks after suture placement (**B**–**D**). (**B**) Spotted expression of Itga-9 at early stage of valve formation. (**C**) Thin ring of Itga-9 expression characteristic of intermediate stage of valve formation. (**D**) Late stage of valve formation identified by strong band of Itga-9 expression, similarly as seen in normal conjunctiva (**A**). Itga-9: red; LYVE-1: green. Original magnification: 400 X. Arrows: lymphatic valves.

Our further examination on morphological structures of corneal lymphatic valves on Day 14 corneas revealed that these valves were presented as band-like structures, similarly as observed in other tissues during development [17]. We also detected various shapes of lymphatic valves from incomplete to more developed ones, possibly indicating an early to late stages of development (Figure 4, B–D). Mature lymphatic valves in the cornea exhibited similar shapes as those found in normal conjunctiva, as demonstrated in Figure 4A.

### Lymphatic valves tend to be located near lymphatic branching points

**Figure 5:**
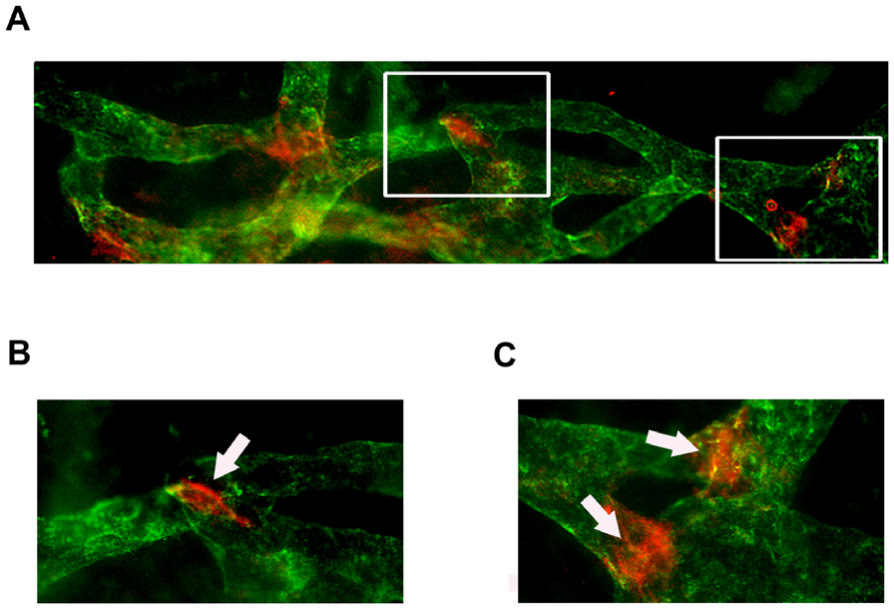
Localization of corneal lymphatic valves at branching points. (**A**–**C**) Representative micrographs demonstrating the location of newly formed lymphatic valves at vessel branching points, as indicated by the arrows. Itga-9: red; LYVE-1: green. (**B** and **C**) Higher magnification views of the boxed areas in (**A**). Original magnification: 200 X (**A**) and 400 X (**B** and **C**).

We next examined the location of the valves along newly formed lymphatic vessels in the inflamed corneas. Interestingly, it was found that the lymphatic valves were more often located at or near the branching points, as demonstrated in Figure 5. The percentage of lymphatic valves around the branching points over the total number of lymphatic valves was 68% in Day 14 corneas. Moreover, we have measured the diameters of corneal lymphatics with valves, which averaged about 24.8 +/− 1.0 µm (SEM). No significant difference was found between the diameters of the lymphatic vessels hosting valves at the branching points or in the middle of the vessels (data not shown).

## Discussion

In this study, we have provided the first evidence showing that newly formed lymphatic vessels in the cornea develop valves. With the progression of corneal inflammatory LG, the lymphatic valve formation increases as well. In addition to defining the time course of this process, we have provided detailed information on the average size of the lymphatic vessels hosting valves, and the likely locations where the valves tend to form. It is logical that lymphatic valves tend to be located near the branching points where lymphatic vessels bifurcate and lymph flow needs to be redirected.

Since lymphatic valve formation occurs after endothelial cells cover the inner surface of lymphatic walls, it serves as a maturation index for LG. Our data showing that inflamed lymphatics of early time points (such as Day 3 and 7) are smaller in size and also not equipped with valves indicate that these lymphatics are still immature, and may be more responsive to drug therapy. Allied to this notion is another recent report from our laboratory showing that corneal LG can be suppressed within a critical time window after the inflammatory stimulation and when a blockade of vascular endothelial growth factor receptors (VEGFR) is initiated

during early- and middle- but not late-stage LG [20].

This study may also provide a new therapeutic target to treat corneal LG and its related diseases. Since lymphatic valves are tightly associated with the functions of the vessels, it is plausible to hypothesize that future strategies targeting on lymphatic valve formation, maturation, or maintenance may impede the function of lymphatic vessels and lead to a better outcome of the diseases. Moreover, we have demonstrated in this study that Itga-9 is up-regulated during corneal LG and lymphatic valve formation. A molecular blockade of the Itga-9 pathway may therefore offer a novel strategy to treat lymphatic-related diseases in the cornea, which warrants further investigation.

Beyond its contribution to corneal research, this study bears broader implications. For

example, it reveals a new *in vivo* model with easy access to a transparent tissue to study pathological lymphatic valve formation, maturation, and manipulation. Results from corneal lymphatic valve studies will be readily applicable to other research fields as well. As proven in the past, the cornea has provided a powerful tool to study a broad spectrum of angiogenic processes, whether they pertain to tumor growth and metastasis, or specific growth factors [6], [23], [24]. As mentioned earlier, a wide array of diseases are associated with lymphatic dysfunction in the body, which can be disabling and even life threatening. It is hopeful that results from this study will shed some light on our understanding of pathological lymphatic valve formation as well as the development of new therapies to treat lymphatic disorders in general.
